# The complete mitochondrial genomes of two erythroneurine leafhoppers (Hemiptera, Cicadellidae, Typhlocybinae, Erythroneurini) with assessment of the phylogenetic status and relationships of tribes of Typhlocybinae

**DOI:** 10.3897/zookeys.1037.63671

**Published:** 2021-05-17

**Authors:** Xiaoxiao Chen, Can Li, Yuehua Song

**Affiliations:** 1 School of Karst Science, Guizhou Normal University/ State Key Laboratory Cultivation Base for Guizhou Karst Mountain Ecology Environment of China, Guiyang, Guizhou 550001, China Guizhou Normal University Guiyang China; 2 Guizhou Provincial Key Laboratory for Rare Animal and Economic Insect of the Mountainous Region, Guiyang University, Guiyang, Guizhou 550001, China Guiyang University Guiyang China

**Keywords:** *Mitjaevia dworakowskae*, *Mitjaevia shibingensis*, mitochondrial genome, phylogenetic analysis, tribal taxonomic status

## Abstract

The number and classification of tribes in the leafhopper subfamily Typhlocybinae are not yet fully clear, and molecular data has recently been used to help resolve the problem. In this study, the mitochondrial genomes of *Mitjaevia shibingensis* Chen, Song & Webb, 2020 and *M. dworakowskae* Chen, Song & Webb, 2020 of the tribe Erythroneurini (Cicadellidae, Typhlocybinae) were sequenced. Most protein-coding genes (PCGs) start with ATN and end with TAA or TAG, and the AT content of these three codons were found differ from previous results that show that the first codon has the highest incidence. Two rRNA genes are highly conserved, and the AT content in *16S* is higher than that of *12S*. The nucleotide diversity and genetic distance among 13 PCGs of the four tribes from Typhlocybinae show that Empoascini nucleotide diversity is significantly less than in the other three tribes, and have the largest distance from the others, while Typhlocybini and Zyginellini have the smallest distance, indicating that the relationship between the two is the closest. The *nad2*, *nad4*, *nad4L*, and *nad5* genes have greater nucleotide diversity, showing potential for use as the main markers for species identification. The phylogenetic analysis yielded a well-supported topology with most branches receiving maximum support and a few branches pertaining to relationships within Zyginellini and Typhlocybini receiving lower support. The species of these two tribes are intertwined, and it was impossible to resolve them into separate branches. In addition, the tribes Empoascini and Erythroneurini were recovered as monophyletic, and Alebrini was placed at the base of the tree as the most primitive. These results are broadly in line with other molecular phylogenetical studies which differ from traditional morphological classification.

## Introduction

Cicadellidae (leafhoppers) are the largest family of the order Hemiptera. Representatives are important agricultural and forestry pests that feed on a variety of plants such as cereal crops, vegetables, and fruit trees, and they are also vectors of plant pathogens (Morris 1971; Guo 2011; Roddee et al. 2018). Leafhoppers also have many interesting characteristics, such as varied lifestyles and feeding strategies (including utilizing different endosymbionts), covering their bodies with brochosomes, and producing courtship signals through the plant substrate. These characteristics make leafhoppers suitable material for studies on biological evolution and geographical research (Chen et al. 2020a, b). The subfamily of Typhlocybinae is the second largest group of Cicadellidae and is widely distributed in the six major zoogeographic regions of the world. Erythroneurini, the largest tribe of Typhlocybinae, includes ~2,000 species worldwide (Wang et al. 2012).

The traditional classification of leafhopper has attracted much research attention, including the classification of Typhlocybinae. At present, Typhlocybinae contains six tribes (Alebrini, Empoascini, Erythroneurini, Zyginellini, Typhlocybini, Dikraneurini) but this division remains controversial (Hamilton 1998; Balme 2007; Dietrich 2013). Today, the emergence of next-generation sequencing technology is a breakthrough for solving this problem enabling mitochondrial genomic data to verify and reference the existing family-level classification of Typhlocybinae. Many previous attempts have been made to estimate phylogenetic relationships among leafhoppers mostly by using either morphological data or sequence data from a few gene regions (Hamilton 1983; Dietrich et al. 2001; Zahniser and Dietrich 2013; Krishnankutty et al. 2016), but there is very little research on Typhlocybinae. So far, in the National Center for Biotechnology Information (NCBI), Typhlocybinae only has complete mitochondrial genomic data for 19 species (Table 1).

The insect mitochondrial genome (mtDNA) is usually a closed double-stranded DNA molecule with a molecular weight of 14–20 kb. Usually, it contains 37 genes, including 13 protein-coding genes (PCGs), NADH dehydrogenase 1-6 and 4L (nad1-6 and *nad4L*), cytochrome c oxidase subunits 1-3 (cox1-3), ATPase subunit 6 and 8 (*atp6* and *atp8*), cytochrome b (*cytb*), two ribosomal RNAs genes (*16S* and *12S*) and 22 transfer RNA (tRNA) genes. A region rich in A + T, the control region, is also present (Boore 1999; Wang et al. 2018). Compared with the nuclear genome, the insect mitochondrial genome has the characteristics of a simple structure, low molecular weight, stable composition, conservative arrangement, maternal inheritance, and easy detection. It is very suitable for the study of evolutionary genomics and is widely used to identify the phylogenetic relationships and population structures at different taxonomic levels (Saccone et al. 2002; Cook et al. 2005; Wilson et al. 2008).

To further enrich the mitochondrial genome data of leafhoppers and provide comparative data for closely related species, we sequenced and analyzed the complete mitochondrial genomes of *Mitjaevia shibingensis* and *M. dworakowskae* and analyzed their phylogenetic relationship with other Typhlocybinae. The new molecular data obtained will help in the identification of leafhopper species, kinship comparison, and future studies on population genetics and evolution.

## Materials and methods

### Mitogenome sequencing, assembly, and annotation

For this study, samples of *Mitjaevia shibingensis* and *M. dworakowskae* were collected in 100% alcohol and stored at –20 °C in the laboratory. Total DNA was extracted from the entire body without the abdomen and wings. The mitochondrial gene sequences were obtained through second-generation sequencing. Primers were designed to amplify the mtDNA sequence in PCR reactions. The PCR reaction was performed using the LA Taq polymerase. The PCR conditions were as follows: initial denaturation at 94 °C for 2 min, then 35 cycles of denaturation at 94 °C for 30 sec, annealing at 55 °C for 30 sec, and extension at 72 °C for 1 min/kb, followed by the final extension at 72 °C for 10 min. The PCR products were sequenced directly, or, if needed, first cloned into a pMD18-T vector (Takara, JAP) and then sequenced, by the dideoxynucleotide procedure, using an ABI 3730 automatic sequencer (Sanger sequencing) using the same set of primers. After quality-proofing of the obtained fragments, the complete mt genome sequence was assembled manually using DNAStar (Burland 2000), and a homology search was performed by the Blast function in NCBI to verify the amplified sequence as the target sequence (Meng et al. 2013; Yu et al. 2017). The nucleotide base composition, codon usage, and A + T content values were analyzed with MEGA 6.06 (Tamura et al. 2013). The secondary structure of tRNA genes was annotated using online tools tRNAscan-SE 1.21 and ARWEN (Lowe and Eddy 1997; Laslett and Canbäck 2008). The tandem repeat sequence in the control area was determined by the online search tool Tandem Repeats Finder (Benson 1999). The base skew values for a given strand were calculated using the formulae: AT skew = [A – T] / [A + T] and GC skew = [G – C] / [G + C] (Perna and Kocher 1995). The nucleotide diversity (Pi) and sliding window analysis (sliding window: 200 bp, step size: 20 bp) of 13 PCGs among four tribes of Typhlocybinae species were conducted with DnaSP 5.0 software (Rozas et al. 2017). And the genetic distance of the four tribes was estimated in MEGA 6.06.

### Phylogenetic analysis

The phylogenetic analysis included two sets of data. First, the phylogenetic tree was constructed based on 29 *cox1* data among six tribes of Typhlocybiane and two outgroups. Secondly, phylogenetic tree analysis was conducted using a dataset including the complete mitochondrial genomes of the two newly sequenced erythroneurine species, 17 typhlocybiane species, and two outgroups, of which nine sets of data were from team sequencing, while the remaining 10 were obtained from the NCBI database (Table 1).

**Table 1.** List of the mitochondrial genomes analyzed in the present study.

Tribe	Species	Length (bp)	GenBank accession no.
Empoascini	*Empoasca flavescens*	15,152	MK211224.1
	*Empoasca onukii*	15,167	NC_037210.1
	*Empoasca vitis*	15,154	NC_024838.1
	*Ghauriana sinensis*	15,491	MN699874.1
Erythroneurini	*Empoascanara dwalata*	15,271	MT350235.1
	*Empoascanara gracilis*	14,627	MT576649
	*Empoascanara sipra*	14,827	NC_048516.1
	*Empoascanara wengangensis*	14,830	MT445764
	*Illinigina* sp.	14,803	KY039129.1
	*Mitjaevia dworakowskae*	16,399	MT981880
	*Mitjaevia protuberanta*	15,472	NC_047465.1
	*Mitjaevia shibingensis*	15,788	MT981879
Typhlocybini	*Bolanusoides shaanxiensis*	15,274	MN661136.1
	*Eupteryx minuscula*	16,944	MN910279.1
	*Typhlocyba* sp.	15,223	KY039138.1
Zyginellini	*Limassolla lingchuanensis*	15,716	MN605256.1
	*Paraahimia luodianensis*	16,497	NC_047464.1
	*Parathailocyba orla*	15,382	MN894531.1
	*Zyginella minuta*	15,544	MT488436.1

The Gblocks Server online platform was used to eliminate poorly aligned positions and divergent regions of DNA protein alignment, and all alignments were checked and corrected in MEGA 6.06 prior to the phylogenetic analysis (Tamura et al. 2013). Five datasets were generated: (1) *cox1* with 573 nucleotides (2) 13 PCGs with 10,452 nucleotides (PCGs); (3) the first and second codon positions of the 13 PCGs with 6968 nucleotides (PCG12); (4) 13 PCGs with 10,452 nucleotides and 2 rRNA with 1615 nucleotides (PCGR); (5) and amino acid sequences of the 13 PCGs with 2666 amino acids (PCGAA).

The trimmed datasets were used to estimate the phylogeny by maximum likelihood (ML) using IQ-TREE and Bayesian inference (BI) using MrBayes 3.2.7 (Zhou et al. 2011; Du et al. 2017). ML constructed with the IQ-TREE used an ultrafast bootstrap approximation approach with 10,000 replicates and calculated bootstrap scores for each node (BP). BI selected GTR + I + G as the optimal model, running 10 million generations twice, sampling once every 1000 generations, after the average standard deviation of the segmentation frequency drops below 0.01, with the first 25% of the samples are discarded burn-in, and the remaining trees used to generate a consensus tree and calculate the posterior probability (PP) of each branch.

## Results and discussion

### Organization and composition of the genome

**Figure 1.**
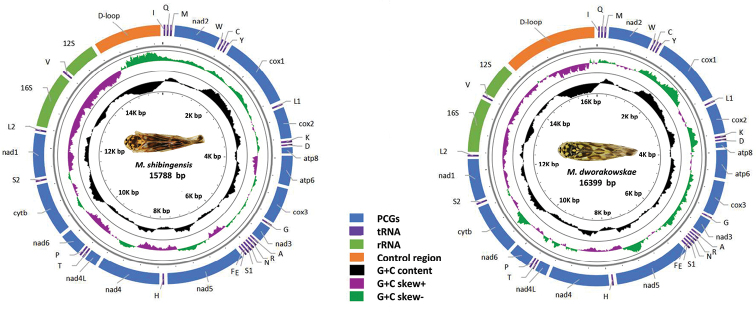
Circular maps of the mitochondrial genome of *Mitjaevia shibingensis* and *M. dworakowskae*.

The genomic organization and nucleotide composition of the two new mitogenomes sequenced in this study are similar to those of other previously reported Typhlocybina (Tan et al. 2020; Yuan et al. 2020). The complete mitogenomes of *M. shibingensis* and *M. dworakowskae* are double-stranded plasmids with 15,788 and 16,399 bp, respectively. Both species contain the usual 13 PCGs, 22 tRNA genes, 2 rRNA genes, and a control region (Fig. 1). Fourteen genes encode in the minority strand (L-strand) while the others encode in the majority strand (H-strand). *Mitjaevia shibingensis* has a total of 46 bp intergenic space in 12 regions ranging from 1 to 8 bp. Eleven genes were found to overlap by a total of 47 bp. *Mitjaevia dworakowskae* has a total of 84 bp intergenic space in 12 regions ranging from 2 to 15 bp, and seven genes were found to overlap by a total of 23 bp (Table 2).

**Table 2.** Organization of the *Mitjaevia shibingensis* and *M. dworakowskae* mitochondrial genome.

*M. shibingensis*/*M. dworakowskae*											
Gene	Position		Size (bp)		Intergenic		Start codon		Stop codon		Strand
tRNA-*Ile*	1–63	1–63	63		0						H
tRNA-*Gln*	61–128	61–128	68		–3	0					L
tRNA-*Met*	151–219	137–205	69		8	9					H
*nad2*	220–1191	206–1177	972		0		ATA		TAA		H
tRNA-*Trp*	1190–1253	1176–1235	64	60	–2	2					H
tRNA-*Cys*	1246–1307	1233–1295	62	63	–8						L
tRNA-*Tyr*	1307–1368	1299–1364	62	66	5	3					L
*cox1*	1378–2913	1374–2909	1536		1	2	ATG		TAA	TAG	H
tRNA-*Leu*	2915–2980	2911–2976	66		0	2					H
*cox2*	2981–3659	2977–3655	679		0		ATT		T		H
tRNA-*Lys*	3660–3730	3656–3726	71		0						H
tRNA-*Asp*	3733–3793	3727–3789	61	63	–1	0					H
*atp8*	3792–3944	3799–3942	153	144	–1	–2	TTG	ATA	TAA		H
*atp6*	3938–4591	3936–4589	654		–7	0	ATG		TAA		H
*cox3*	4592–5371	4590–5369	780		2	8	ATG		TAA		H
tRNA-*Gly*	5376–5437	5370–5431	62		0						H
*nad3*	5438–5791	5432–5785	354		0		ATT	ATA	TAA		H
tRNA-*Ala*	5796–5857	5791–5855	62	65	4	–2					H
tRNA-*Arg*	5857–5921	5861–5922	65	62	2	15					H
tRNA-*Asn*	5912–5986	5922–5981	66	60	–2	0					H
tRNA-*Ser*	5986–6053	5986–6052	68	67	–4	–1					H
tRNA-*Glu*	6055–6118	6058–6123	64	66	8	11					H
tRNA-*Phe*	6135–6197	6128–6195	63	68	4	2					L
*nad5*	6200–7873	6200–7873	1674		0		TTG		TAA		L
tRNA-*His*	7874–7937	7874–7937	64		0						L
*nad4*	7937–9265	7937–9265	1329		–7	–1	ATG		TAA		L
*nad4L*	9259–9537	9259–9537	279		1	–7	ATG		TAA		L
tRNA-*Thr*	9540–9605	9540–9604	66	65	2	0					H
tRNA-*Pro*	9606–9671	9605–9671	66	67	0	7					L
*nad6*	9674–10159	9674–10159	486		2	5	ATT		TAA		H
*cytb*	10166–11302	10162–11298	1137		7	0	ATG		TAG	TAA	H
tRNA-*Ser*	11309–11372	11298–11363	64	66	–2	9					H
*nad1*	11363–12304	11366–12296	942	931	–10	–2	ATT		TAA	T	L
tRNA-*Leu*	12305–12370	12297–12364	66	68	0						L
*16S*	12371–13562	12365–13549	1192	1185	0						L
tRNA-*Val*	13563–13627	13550–13615	65	66	0						L
*12S*	13628–14359	13616–14351	732	736	0						L
D-loop	14360–15788	14352–16399	1429	2048							

**Figure 2.**
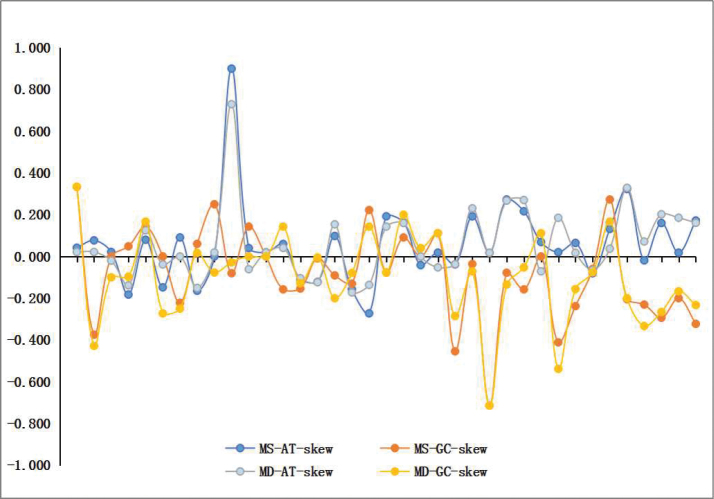
AT and GC skews calculated for the 37 mitochondrial genomes of *Mitjaevia shibingensis* and *M. dworakowskae*. Each point indicates an individual gene.

The AT contents and skew statistics are shown in Table 3. The mitochondrial genomes of *M. shibingensis and M. dworakowskae* show heavy AT nucleotide bias, with A + T% content for the whole sequence was 78.4% and 79.0%, respectively. Similar patterns of nucleotide composition are also found in other leafhopper species (Du et al. 2017; Wang et al. 2018; Xian et al. 2020). The control region (CR) has the strongest A + T% bias, while the PCGs shows the lowest A + T% among whole genes. The whole genome has positive AT skews (0.042, 0.051) and negative GC skews (–0.074, –0.104). Analysis of 37 individual genes of the two species show that AT skews are mostly positive, while for GC skews, the genes of both species are mostly negative (Fig. 2). Positive AT skews indicates that the content of base A is higher than that of base T. However, in a few genes, although the AT skews is negative, the difference in absolute value was very small. For negative GC skew, a negative value indicates that the content of base G is lower than that of base C, while a positive value indicates the opposite. In general, the basic composition of these two species is biased towards A and C.

**Table 3.** Nucleotide compositions, AT skew, and GC skew in different regions of *Mitjaevia shibingensis* and *M. dworakowskae* mitochondrial genomes.

Feature	A%	C%	G%	T%	A+T%	AT skew	GC skew	Length (bp)
***M. shibingensis***								
Whole	40.8	11.6	10.0	37.6	78.4	0.042	–0.074	15,788
PCGs	39.3	12.8	11.5	36.4	75.7	0.038	–0.053	10975
1^st^ codon position	41.6	12.1	11.5	34.9	76.5	0.087	–0.026	3659
2^nd^ codon position	38.3	12.2	11.7	37.8	76.1	0.007	–0.018	3658
3^rd^ codon position	38.0	14.1	11.3	36.6	74.5	0.019	–0.111	3658
tRNA	40.6	10.9	10.1	38.4	79.0	0.028	–0.037	1427
16S	48.1	11.1	6.0	34.8	82.9	0.160	–0.294	1192
12S	48.0	12.0	6.1	33.9	81.8	0.172	–0.323	732
CR	42.7	3.5	3.8	50.0	92.7	–0.079	0.038	1429
***M. dworakowskae***								
Whole	41.5	11.6	9.4	37.5	79.0	0.051	–0.104	16,399
PCGs	40.1	12.5	10.8	36.6	76.7	0.046	–0.073	10955
1^st^ codon position	42.4	11.9	10.6	35.1	77.5	0.095	–0.055	3652
2^nd^ codon position	38.0	12.9	11.7	37.3	75.4	0.009	–0.047	3652
3^rd^ codon position	39.9	12.7	10.0	37.4	77.3	0.032	–0.119	3651
tRNA	40.6	11.6	9.9	37.9	78.5	0.034	–0.081	1435
16S	49.6	11.1	6.4	32.9	82.5	0.202	–0.266	1185
12S	47.6	11.1	6.9	34.4	81.9	0.161	–0.233	736
CR	43.1	6.9	4.2	45.8	88.9	–0.030	–0.246	2048

### Protein-coding genes and codon usage

Similar to other Typhlocybinae mitochondrial genomes, of the 13 PCGs of *M. shibingensis and M. dworakowskae*, nine genes (*cox1*, *cox2*, *cox3*, *atp8*, *atp6*, *nad2*, *nad3*, *nad6*, and *cytb*) are located on the major strand (H-strand) while the other four PCGs (*nad4*, *nad4L*, *nad5*, and *nad1*) are located on the minor strand (L-strand). The largest gene was the *nad5* gene, and the smallest was the *atp8* gene in erythroneurine mitogenomes. The average AT content values of PCGs were 75.4% and 76.7% in *M. shibingensis and M. dworakowskae*, respectively. The A + T content of the first codon positions (76.5%, 77.5%) was much higher than that of the second (76.1%, 75.4%) and the third (74.5%, 77.3%) positions. This result was different from most other studies which show that the third codon has the highest AT content (Du et al. 2019; Yang et al. 2020). AT skews of all codon positions is positive while GC skews are negative. Most PCGs have the standard ATN (ATA/ATT/ATG/ATC) as the start codon, while *nad5* and *atp8* genes have TTG, a pattern also observed in other leafhopper mitogenomes (Wang et al. 2017; Wang et al. 2020). Conventional stop codons (TAA or TAG) appear in most PCGs, except that *cox2* and *nad1* use an incomplete codon (a single T--) as the stop codon (see Tables 2, 3).

**Figure 3.**
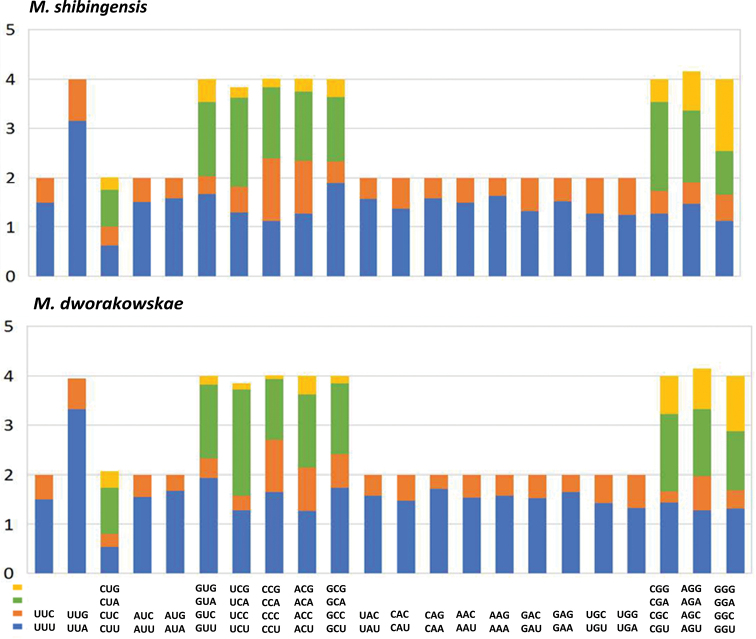
Relative Synonymous Codon Usage (RSCU) of mitochondrial genomes for *Mitjaevia shibingensis* and *M. dworakowskae*.

Research determined the behavior of the PCG codon families and found that codon usage was very similar among Cicadellidae mitogenomes when the results of two species were calculated and summarized (see Table 4, Fig. 3). All 62 available codons (excluding TAA and TAG) are present in *M. shibingensis and M. dworakowskae*. Synonymous codon usage bias was observed in both mitochondrial genomes, and 22 codons were used more frequently than other codons. The four most abundant codons were AAU (Asn), AAA (Lys), AUU (Ile), and UUA (Leu2). The preferred codons all end with A or U, which contribute to the high A + T bias of the entire mitogenomes.

**Table 4.** Codon and Relative Synonymous Codon Usage (RSCU) of 13 PCGs in the mt genomes of *Mitjaevia shibingensis* and *M. dworakowskae*.

Amino acid	* Codon *	*Count/RSCU*				Amino acid	* Codon *	*Count/RSCU*			
		*M. shibingensis*		*M. dworakowskae*				*M. shibingensis*		*M. dworakowskae*	
Phe	UUU	**190** ^a^	**1.49**	**200**	**1.5**	Tyr	UAU	**190**	**1.56**	**185**	**1.57**
	UUC	65	0.51	66	0.5		UAC	53	0.44	51	0.43
Leu2	UUA	**234**	**3.15**	**245**	**3.33**	His	CAU	**57**	**1.37**	**49**	**1.48**
	UUG	63	0.85	45	0.61		CAC	26	0.63	17	0.52
Leu1	CUU	**46**	**0.62**	39	0.53	Gln	CAA	**56**	**1.58**	**66**	**1.71**
	CUC	28	0.38	20	0.27		CAG	15	0.42	11	0.29
	CUA	56	0.75	**69**	**0.94**	Asn	AAU	**269**	**1.49**	**281**	**1.54**
	CUG	19	0.26	24	0.33		AAC	91	0.51	84	0.46
Ile	AUU	**226**	**1.5**	**225**	**1.55**	Lys	AAA	**242**	**1.62**	**246**	**1.57**
	AUC	75	0.5	66	0.45		AAG	56	0.38	67	0.43
Met	AUA	**201**	**1.58**	**213**	**1.68**	Asp	GAU	**35**	**1.32**	**35**	**1.52**
	AUG	54	0.42	40	0.32		GAC	18	0.68	11	0.48
Val	GUU	**52**	**1.66**	**53**	**1.93**	Glu	GAA	**56**	**1.51**	**62**	**1.65**
	GUC	12	0.38	11	0.4		GAG	18	0.49	13	0.35
	GUA	47	1.5	41	1.49	Cys	UGU	**27**	**1.26**	**37**	**1.42**
	GUG	14	0.45	5	0.18		UGC	16	0.74	15	0.58
Ser2	UCU	47	1.29	48	1.28	Trp	UGA	**49**	**1.24**	**54**	**1.33**
	UCC	19	0.52	11	0.29		UGG	30	0.76	27	0.67
	UCA	**66**	**1.81**	**81**	**2.15**	Arg	CGU	14	1.27	13	1.44
	UCG	8	0.22	5	0.13		CGC	5	0.45	2	0.22
Pro	CCU	26	1.11	**42**	**1.65**		CGA	**20**	**1.82**	**14**	**1.56**
	CCC	30	1.28	27	1.06		CGG	5	0.45	7	0.78
	CCA	**34**	**1.45**	31	1.22	Ser1	AGU	**53**	**1.46**	48	1.28
	CCG	4	0.17	2	0.08		AGC	16	0.44	26	0.69
Thr	ACU	49	1.26	54	1.26		AGA	**53**	**1.46**	**51**	**1.36**
	ACC	42	1.08	38	0.89		AGG	29	0.8	31	0.82
	ACA	**55**	**1.41**	**63**	**1.47**	Gly	GGU	31	1.11	**34**	**1.31**
	ACG	10	0.26	16	0.37		GGC	15	0.54	10	0.38
Ala	GCU	**26**	**1.89**	**23**	**1.74**		GGA	25	0.89	31	1.19
	GCC	6	0.44	9	0.68		GGG	**41**	**1.46**	29	1.12
	GCA	18	1.31	19	1.43	*	UAA	**177**	**1.61**	**170**	**1.61**
	GCG	5	0.36	2	0.15		UAG	43	0.39	41	0.39

^a^ The higher values of preferentially used codons are given in bold.

### Transfer RNA and ribosomal RNA genes

**Figure 4.**
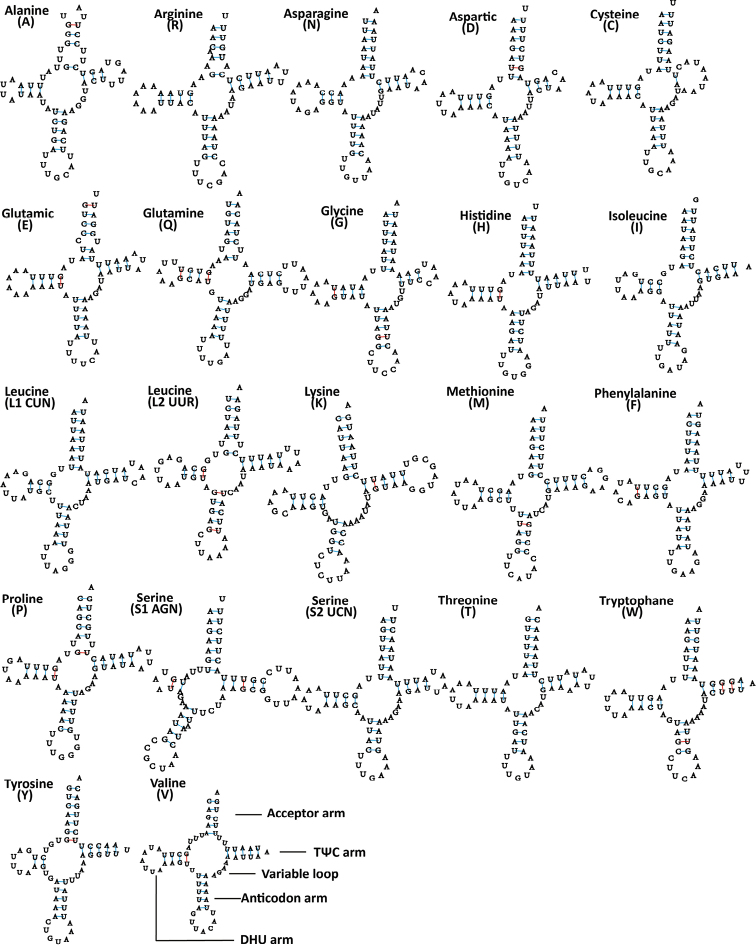
Inferred secondary structures of 22 tRNAs from *Mitjaevia shibingensis*. Watson-Crick base pairings are illustrated by lines (-), whereas GU base pairings are illustrated by red lines (-). Structural elements in tRNA arms and loops are illustrated as for *trnV*.

**Figure 5.**
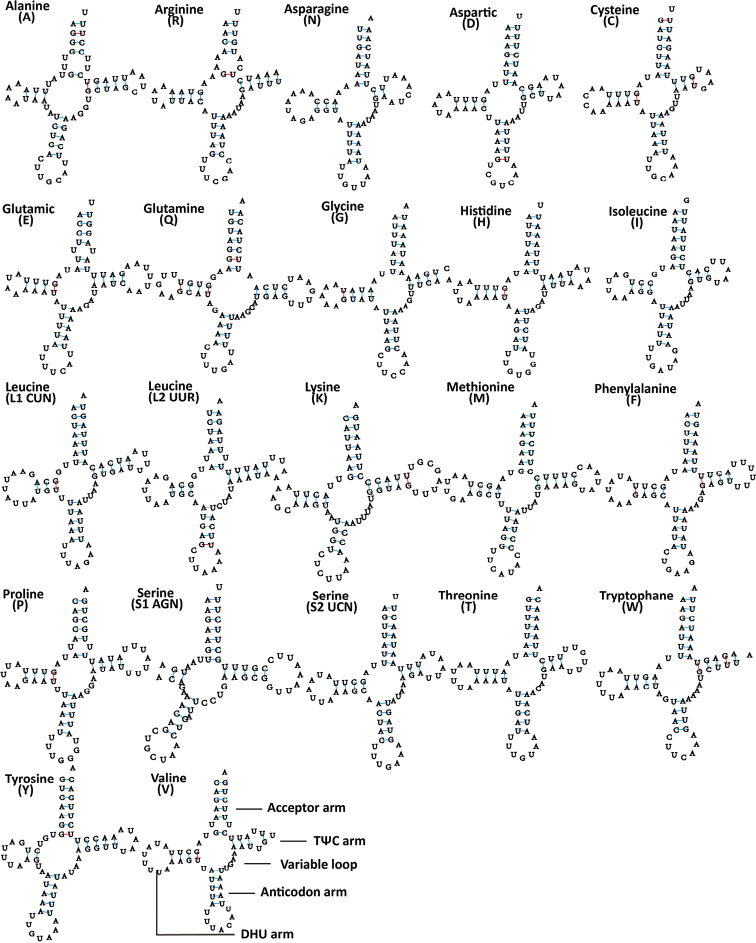
Inferred secondary structures of 22 tRNAs from *Mitjaevia dworakowskae*. Watson-Crick base pairings are illustrated by lines (-), whereas GU base pairings are illustrated by red lines (-). Structural elements in tRNA arms and loops are illustrated as for *trnV*.

All 22 typical tRNA genes are present in the *M. shibingensis* and *M. dworakowskae* mitochondrial genomes, of which 14 genes were oriented on the major strand (H-strand), whereas the others were transcribed on the minor strand (L-strand). Their nucleotide lengths are almost identical between species, ranging from 60 bp to 71 bp (Table 2). The average AT content values of tRNAs is 78.5% and 79% in each species, respectively, and the tRNA genes have negligible AT and GC skews (Table 3). Compared to the ancestral insect mitochondrial gene order, no tRNA gene rearrangements were found. All of the tRNA genes can be folded into typical clover-leaf secondary structures except for the *trnS1* in both species’ mitochondrial genomes, which lacks the dihydrouridine (DHU) stem and forms a simple loop. In the metazoan mt genome, lack of the DHU arm was very common in *trnS1* (Yang et al. 2020). Based on the secondary structure, a total of 24 and 22 G-U weak base pairs were found in tRNAs of *M. shibingensis* and *M. dworakowskae*, respectively (Figs 4, 5). Most mismatched nucleotides were G-U pairs, which form weak bonds in tRNA and non-classical pairs in tRNA secondary structure, similar to other Cicadellidae (Jia et al. 2010).

Leafhopper ribosomal RNA (rRNA) includes *16S* RNA and *12S* RNA. These two genes are highly conserved and are encoded on the minor strand (L-strand). Similar to other known insects, the content of A + T% in *16S* was higher than that of *12S*. The *16s* genes of *M. shibingensis* and *M. dworakowskae* were 1192 bp and 1852 bp in length, with AT contents of 82.90% and 82.50%, respectively, and located between *trnL2* and *trnV*. The *12S* rRNA genes of both were 732 bp and 736 bp in length, with AT contents of 81.80% and 81.90%, respectively, and located after *trnV*. The rRNA genes showed a positive AT skew and negative GC skew (Table 3).

### Control region

**Figure 6.**
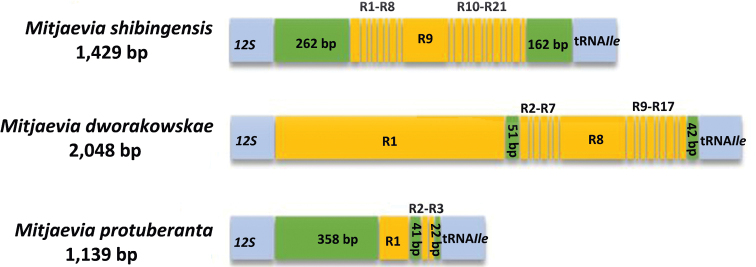
Organization of the control region structure in the mitochondrial genomes of three *Mitjaevia* species. R: repeat unit.

Like the typical insect mitochondrial genome, the mt genomes of *M. shibingensis* and *M. dworakowskae* have a large non-coding region, which was identified as the control region and located downstream of *12S*. Control regions of both species were rich in AT, with lengths of 1429 bp and 2048 bp AT contents of 92.7% and 88.9%, respectively (Table 3). The control regions in the three available *Mitjaevia* mitogenomes were variable and not highly conserved, and their lengths ranged from 15 and 784 bp with variable numbers of repeat sequences (Fig. 6). *Mitjaevia shibingensis* included 21 types of repeat unit (R), 17 kinds of repeats (R1, R2) were found in *M. dworakowskae* with various lengths and copy numbers, and three repeat units were present in *M. protuberanta*. At present, we were unable to find any correlation in repeating units in the different species, probably because of the limited number of species analyzed in this study. Further comparative studies of additional leafhopper mitogenomes are needed.

### Nucleotide diversity and genetic distance analysis

**Figure 7.**
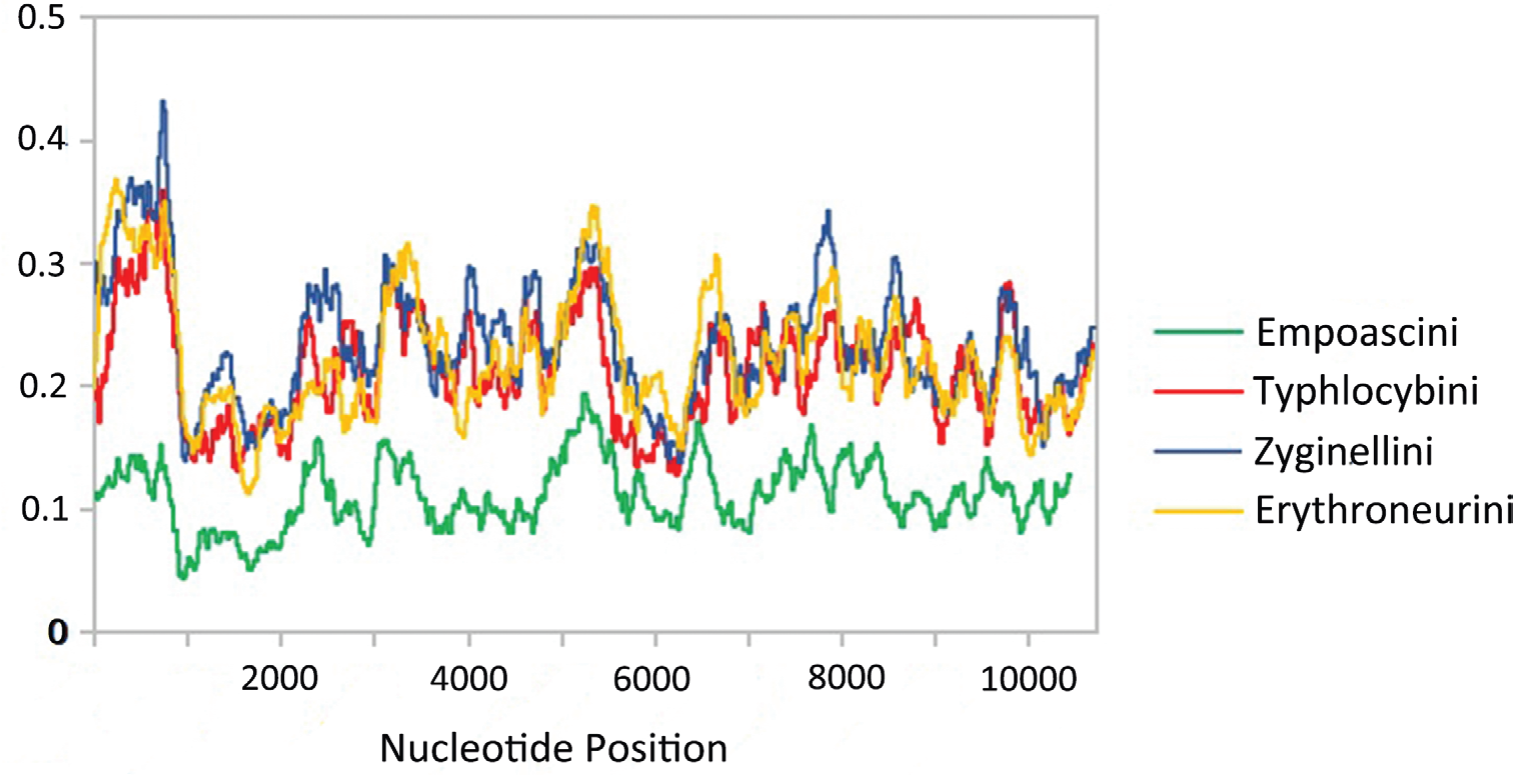
Nucleotide diversity (Pi) and sliding window analysis of 13 PCGs of the four tribes of Typhlocybinae.

The sliding window analysis shows highly variable nucleotide diversity (Pi values) among 13 PCGs sequences of the four tribes of Typhlocybinae (Fig. 7). Empoascini nucleotide diversity is significantly lower than of the other three tribes. The genes *nad2*, *nad4*, *nad4L*, and *nad5* had higher nucleotide diversity, while the genes *cox3*, *cox2*, *cytb*, and *cox1* had comparatively low nucleotide diversity when using MEGA 6.06 software, based on Kimura-2 Parameter, and Bootstrap resampling 1000 times to test and analyze the genetic distance of the four tribes of Typhlocybinae. The results show that the genetic distance between Empoascini and the other three tribes is the largest, and between Typhlocybini and Zyginellini are the smallest (Table 5).

**Table 5.** The genetic distance between the four tribes of Typhlocybinae.

	** Empoascini **	** Typhlocybini **	** Zyginellini **
Typhlocybini	0.3663		
Zyginellini	0.3645	0.2663	
Erythroneurini	0.3623	0.3288	0.3262

Nucleotide diversity analysis, a primary method for identifying the regions with large nucleotide divergence, is especially useful for designing species-specific markers. These are useful for taxa with highly variable morphological characteristics, especially Typhlocybinae species which belong to groups that are difficult to distinguish by morphology alone (Jia et al. 2010; Ma et al. 2019). Among the four tribes, *nad2*, *nad4*, and other highly variable genes have garnered our attention. Whether they can be used as the main marker for species identification or the main related genes that control the appearance of the subfamily is worthy of further study. The genetic distance reflects the distance of the genetic relationships between each tribe. Among the four tribes, Typhlocybini and Zyginellini have the smallest genetic distance, indicating that the relationship between the two is the closest, which is consistent with the results of morphological studies (Zhang 1990; Huang 2013).

### Phylogenetic relationships

#### Historical review

**Figure 8.**
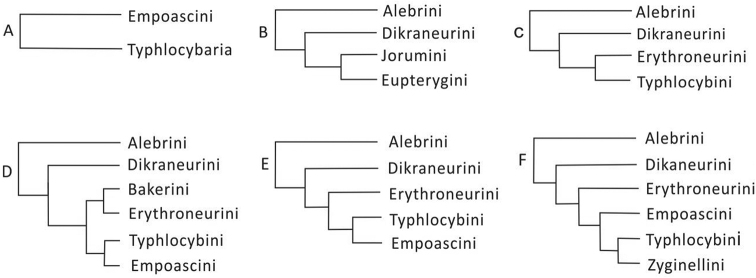
The traditional classification process of tribe levels in the subfamily Typhlocybinae.

Typhlocybinae has been divided into tribes based mainly on the characteristics of the wing veins for the past 90 years. Melichar (1903), Distant (1908, 1918), and Matsumura (1931), among others, divided Typhlocybinae into Empoascaria and Typhlocybaria according to whether the hindwing apical cell is closed (Fig. 8A). McAtee (1934) further divided Typhlocybinae into four tribes, Alebrini, Dikraneurini, Jorumini, and Eupterygini (Fig. 8B), based on the wing veins. Young (1952), also using the male genitalia, recognised the tribes Alebrini, Dikraneurini, Erythroneurini, and Typhlocybini (Fig. 8C). According to whether the peripheral vein of the hind wings exceeds the end of the R+M vein, it was believed that Erythroneurini evolved from Dikraneurini but Mahmood (1967), when adding the tribe Bakerini, believed this condition to be an acquired mutation. At the same time, Mahmood (1967) postulated that Erythroneurini might be more closely related to Typhlocybini, but the relationship between Erythroneurini and the other tribes still need to be determined by studying a large number of specimens. Mahmood and Ahmed (1968a), using the characteristic of the peripheral vein of the hind wings extending to the end of the R vein, separated Empoascini from the former Typhlocybini, and Typhlocybinae was divided into six tribes (Fig. 8D). Dworakowska (1970) compared the characteristics of Bakerini, Typhlocybini, and Erythroneurini, and postulated that Bakerini may be a relatively primitive branch of Erythroneurini (Fig. 8E). Dworakowska (1977, 1990) discussed Typhlocybini with respect to other tribes, and postulated that the different connection modes of the hindwing peripheral vein and CuA represented different branches, and divided Zyginellini from Typhlocybini resulting in six tribes: Alebrini, Dikraneurini, Empoascini, Erythroneurini, Typhlocybini, and Zyginellin, while Zhang (1990) postulated that Erythroneurini evolved from Empoascini. Since then, Typhlocybinae-related research has followed Dworakowska’s six-tribe classification system (Fig. 8F). However, Dietrich (2013) found that the hind wings of leafhoppers have both Zyginellini and Typhlocybini hindwing characteristics when studying the leafhoppers in South America and postulated that the venation characters may not be a stable feature for classification. In terms of overall morphology, Zyginellini and Typhlocybini have similarities present in certain genera and species. Therefore, there is no clear and strong evidence at present to determine whether or not Zyginellini belongs to a natural monophyletic group, and its taxonomic status needs to be further clarified, probably with molecular data.

#### More recent studies

In recent years, molecular sequencing technology has been widely used in phylogenetic analysis, which can test and verify the results of different levels of more morphology based traditional classifications. Within Typhlocybinae, only a few studies have used the combination of morphological characteristics and molecular data to construct phylogenetic relationships. The amount of data is sparse at present and further data is needed.

Dietrich and Dmitriev (2006) used PAUP 4.0b10 to analyze the phylogeny of Typhlocybinae for the first time based on morphological characteristics and concluded that Erythroneurini and Dikraneurini are closely related. However, their analysed samples came mainly from the New World, and whether their results represent the relationship between the tribes of Typhlocybinae remains to be clarified. Balme (2007) combined morphological characteristics with molecular characteristics (*16S* rDNA, *H3*) to perform a phylogenetic analysis of Typhlocybinae, and obtained the following topological structure: Alebrini + ((Empoascini + Typhlocybini) + Dikraneurini), but due to the small sample, the results need to be verified.

Jiang (2016) used *28S* rDNA D2–D3, *16S* rDNA sequence and morphological characteristics to make a preliminary exploration of the phylogenetic relationships of Typhlocybinae and obtained the following topological structure: Alebrini + (Empoascini + Erythroneurini), implying that Erythroneurini is a monophyletic group. Song et al. (2020) constructed a phylogenetic tree of Typhlocybinae using 13PCGs of eight species and obtained the following topology: Empoascini+ (Typhlocybini+ (Erythroneurini+Zyginellini)). These four tribes are all monophyletic, and Erythroneurini and Zyginellini are sister groups, differing only slightly from the traditional morphological classification. Jiao (2017) used MP and NJ methods to analyze the phylogenetic relationship between Alebrini and Dikraneurini based only on morphological data. The results showed that the two tribes’ monophyly was well supported, and its position in the evolutionary tree was similar to that of Zhang (1990) and showed that Alebrini is more primitive.

## Results

This study, based on 29 species of *cox1*, 19 species of 13 PCGs, and two rRNA mitochondrial genes data of Typhlocybinae produced a slightly different result to the traditional classification with respect to Typhlocybini and Zyginellini. Maximum Likelihood (ML) method was used with IQ-TREE using an ultrafast bootstrap approximation approach with 10,000 replicates. The Bayesian Inference (BI) analysis was performed using MrBayes 3.2.7, with the best fit model GTR+I+ G (Vogler and DeSalle 1993).

**Figure 9.**
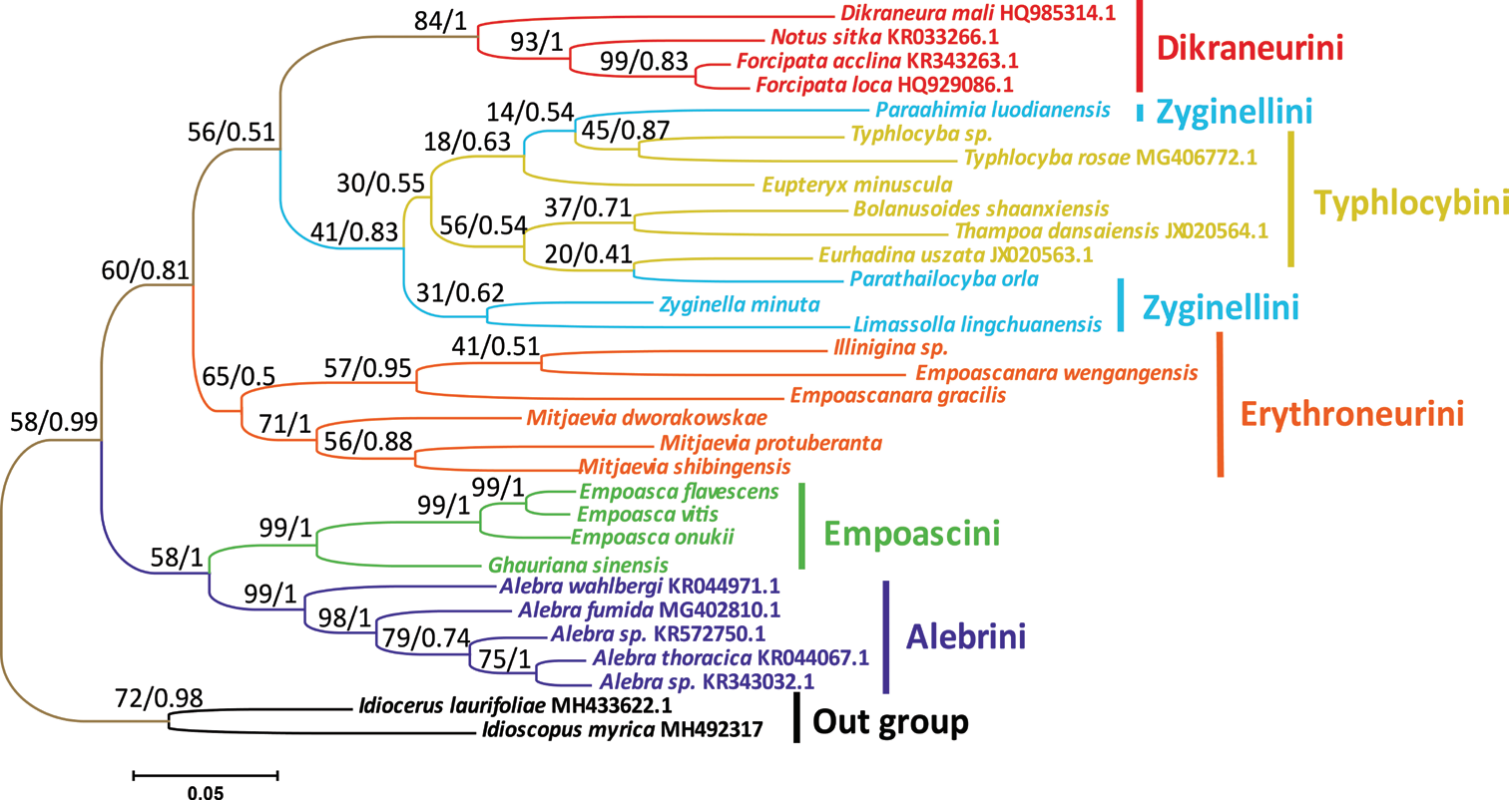
ML and BI Phylogenetic tree inferred from *cox1* of Typhlocybinae. The first number at each node is a bootstrap proportion (BP) of maximum likelihood (ML) analyses, and the second number is Bayesian posterior probability (PP).

*Cox1* is one of the mitochondrial protein-coding genes and its bi-terminal sequence is more conservative than *cox2*. It has a rapid evolution rate and large differences between species, and can provide rich phylogenetic information, hence is an ideal mitochondrial molecular marker. The gene sequences were obtained in the current study by downloading the *cox1* gene sequence of 29 species of Typhlocybinae and two outgroups of Idiocerinae from NCBI to construct a phylogenetic tree. BI and ML analyses generated the same tree topology: (Alebrini + Empoascini) + (Erythroneurini + ((Zyginellini + Typhlocybini) + Dikraneurini))). Most relationships were highly supported, and a few branches pertaining to relationships within Zyginellini and Typhlocybini received lower support (Fig. 9). Also, the tree topology is different from previous research, with Alebrini + Empoascini forming sister groups, and the species of Zyginellini and Typhlocybini are interconnected and cannot be resolved into separate branches. The remaining tribes are monophyletic groups. Alebrini is placed at the base of the tree and is therefore the most primitive. The phylogenetic relationship is generally consistent with the results of previous studies based on morphology and molecules (Balme 2007; Jiang 2016).

**Figure 10.**
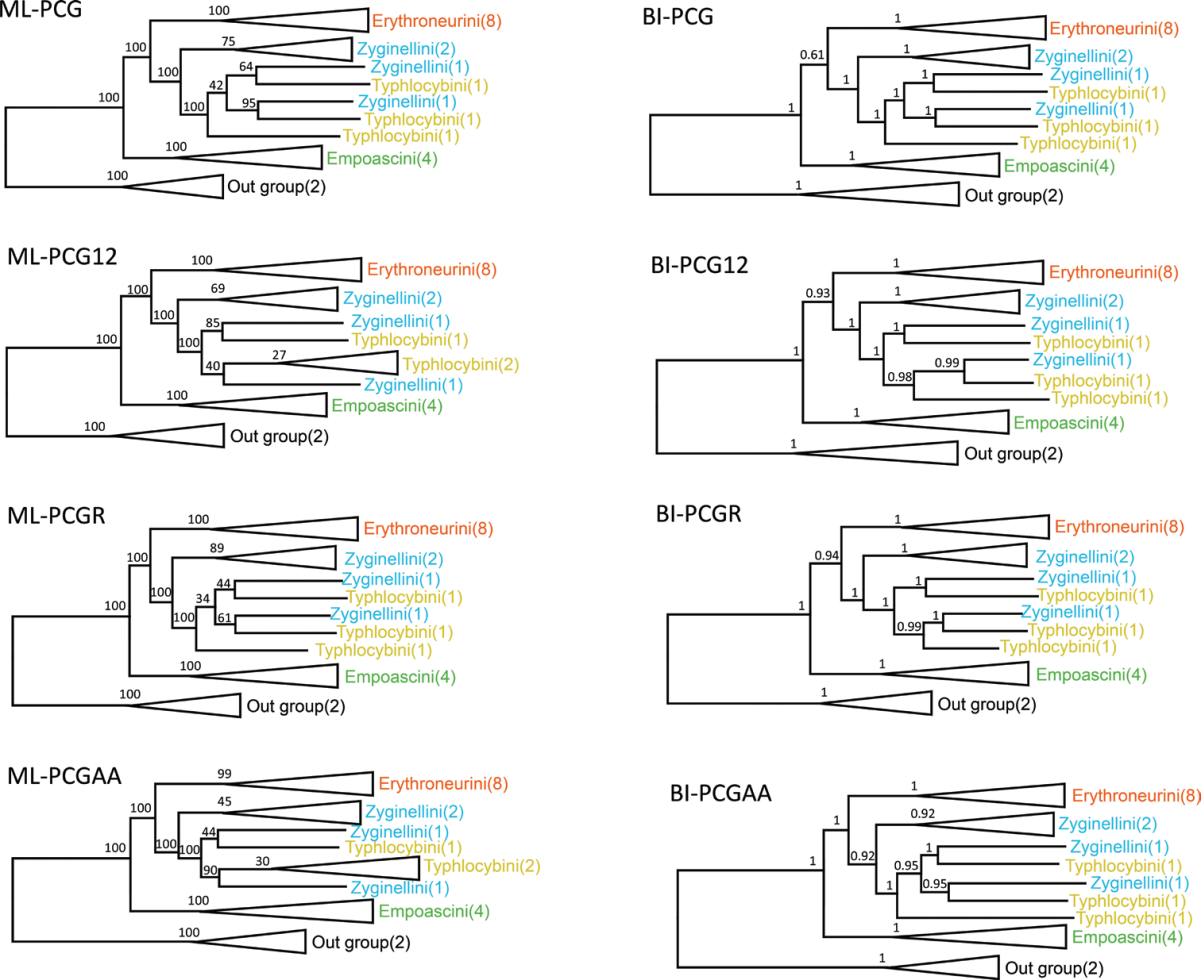
Phylogenetic trees of Typhlocybinae inferred by maximum likelihood (ML) and PhyloBayes (PB) methods based on 13 PCGs and 2 rRNA.

**Figure 11.**
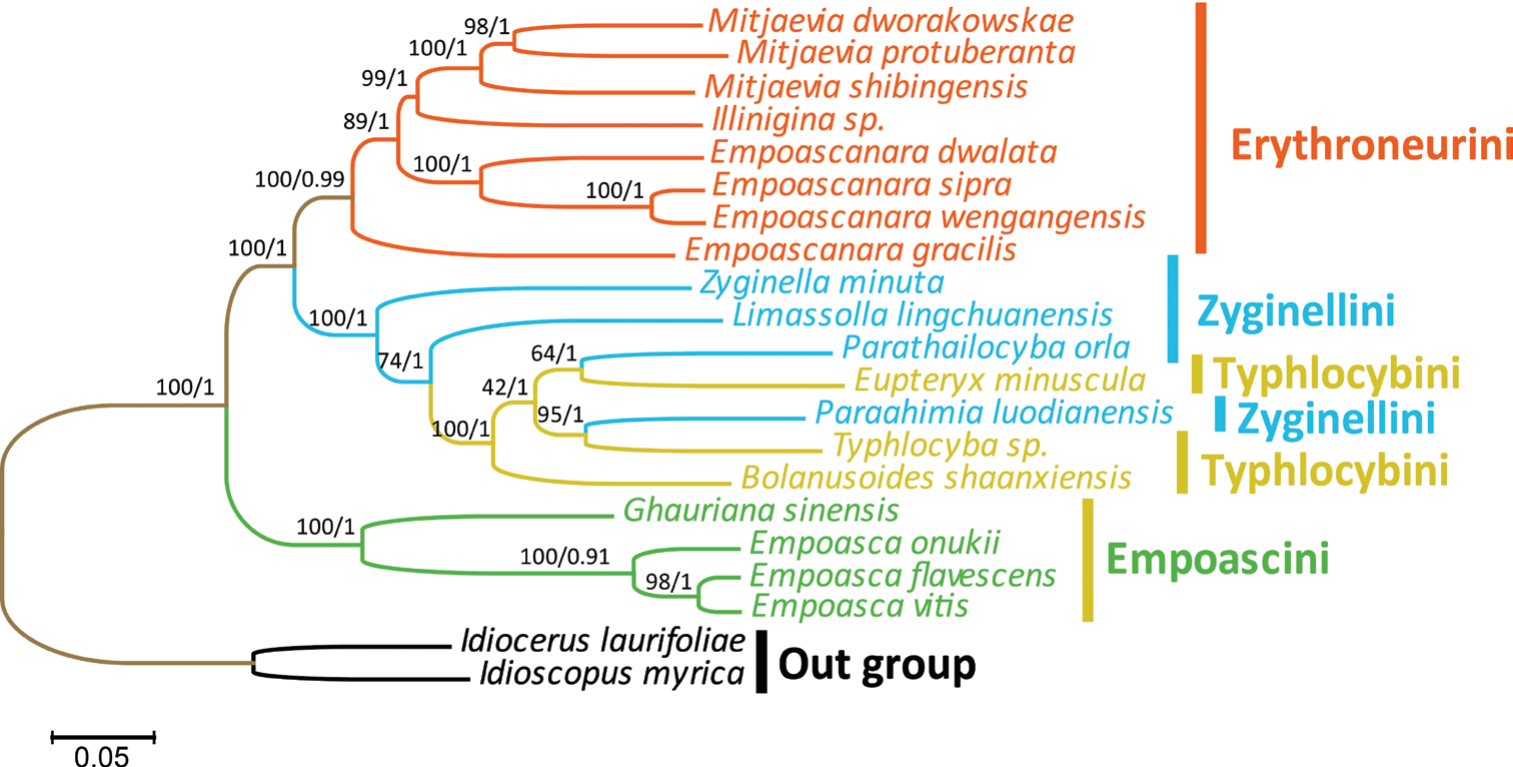
ML and BI Phylogenetic tree inferred from 13 PCGs of Typhlocybinae. The first number at each node is a bootstrap proportion (BP) of maximum likelihood (ML) analyses, and the second number is Bayesian posterior probabilities (PP).

At present, the complete mitochondrial genome data of Alebrini and Dikraneurini have not been added to NCBI. Thus, the phylogenetic relationships were analyzed based on the concatenated nucleotide sequences of 13 PCGs and two rRNA from 19 Typhlocybinae (the remaining four tribes) species and two outgroups. Although ML and PB analyses produced inconsistent topologies across the different datasets and models, most relationships were highly supported and consistent in the analyses, and the main difference is the relationship of species between Zyginellini and Typhlocybini. (Figs 10, 11). In this study, Empoascini and Erythroneurini were recovered as monophyletic, always forming a clade with high support values, while Zyginellini and Typhlocybini formed a single branch in every tree, and neither tribe was ever resolved as monophyletic, which suggests that the hind wing character traditionally used to separate these two tribes is not reliable and that the tribes should probably be treated as synonyms, as was suggested previously by Dietrich (2013). Within the Typhlocybinae, the four species of Empoascini studied constituted one clade and tended to be placed at the basal position of the tree as the sister group to the other tribes. Unlike the previous analyses, the our results support the combination of Zyginellini and Typhlocybini as a tribe. As with other recent studies, our results indicate that sequence data from leafhopper mitogenomes is informative of phylogenetic relationships in the taxonomic hierarchy of this group. Also, the results of the phylogenetic tree and nucleotide diversity are consistent. Empoascini has the lowest nucleotide diversity and is clearly distinguished from the other three tribes. Therefore, we speculate that the richness of nucleotide diversity has an impact on the phylogenetic relationship of Typhlocybinae. However, data are available for only a tiny fraction of species so the addition of more species, and representatives of other major lineages, will be needed to determine the extent to which mitogenome sequence data can resolve leafhopper phylogeny.

## Conclusions

This paper describes the complete mitochondrial genomes of *M. shibingensis* and *M. dworakowskae*, analyzes the basic composition, location, secondary structure, and other characteristics of PCGs, tRNA genes, rRNA genes, and control regions, and compares them to other Typhlocybinae mitochondrial genomes. The mitogenomes of these two species closely resemble those of most other sequenced leafhoppers in various structural and compositional aspects. The sliding window analysis shows a highly variable nucleotide diversity (Pi values) among 13 PCGs sequences of the four tribes of Typhlocybinae. Empoascini nucleotide diversity is significantly lower than in the other three tribes, and the other three tribes have little difference between them. The genes *nad2*, *nad4*, *nad4L*, and *nad5* have higher nucleotide diversity, and whether they can be used as the main markers for species identification or the main related genes that control the appearance of the subfamily is worthy of further study. The genetic distance of the four tribes of Typhlocybinae shows that the Empoascini and the other three tribes are the largest while Typhlocybini and Zyginellini are the smallest and indicates that the relationship between the two is the closest, which is consistent with the results of morphological studies. Phylogenetic analysis of 31 *cox1* yielded a well-supported topology with most branches receiving maximum support and a few branches pertaining to relationships within Zyginellini and Typhlocybini receiving lower support; the species of these two tribes are intertwined and cannot be resolved into separate branches, and Alebrini is placed at the base of the tree as the most primitive. Phylogenetic relationships were analyzed based on the concatenated nucleotide sequences of 13 PCGs and two rRNA show that although ML and PB analyses produced inconsistent topologies across the different datasets and models, and most relationships were highly supported and constant in the analyses. In this study, Empoascini and Erythroneurini were recovered as monophyletic while Zyginellini and Typhlocybini gathered into a single branch and Empoascini tended to be placed at the basal position of the tree as the sister group to the other tribes. This study indicated that mitochondrial genome sequences are informative for leafhopper phylogeny, but unlike the previous analysis (Zhang 1990), the results of this study relocated the taxonomic status and phylogenetic relationship of the six tribes of Typhlocybinae and supported the combination of Zyginellini and Typhlocybini as a single tribe. Also, the results of the phylogenetic tree and nucleotide diversity are consistent. Empoascini has the lowest nucleotide diversity and is clearly distinguished from the other three tribes. Therefore, we speculate that the richness of nucleotide diversity has an impact on the phylogenetic relationship of Typhlocybinae.

Based on the current and previous studies, the classification of the tribes of Typhlocybinae is not yet fully resolved with respect to Typhlocybini and Zyginellini, i.e., one or two tribes. From a molecular perspective, more sequencing data is needed to build a more complete phylogenetic tree to support or modify the traditional morphological classification. To this aim, it is hoped that the new data provided here will facilitate future comparative studies of leafhopper mitogenomes and demonstrate the need for more comparative data.

## Supplementary materials

Supplementary material 1 Datasets of phylogenetic analysis Data type phylogenetic https://binary.pensoft.net/file/543875This dataset is made available under the Open Database License (http://opendatacommons.org/licenses/odbl/1.0/). The Open Database License (ODbL) is a license agreement intended to allow users to freely share, modify, and use this Dataset while maintaining this same freedom for others, provided that the original source and author(s) are credited.Xiaoxiao Chen, Can Li, Yuehua Song
